# A new perspective during laryngo-tracheal surgery: the use of an ultra-thin endotracheal tube (Tritube®) and flow-controlled ventilation—a retrospective case series and a review of the literature

**DOI:** 10.1186/s44158-022-00066-3

**Published:** 2022-08-26

**Authors:** Alberto Grassetto, Tommaso Pettenuzzo, Flavio Badii, Francesca Barzaghi, Riccardo Carlon, Sandro Dellarole, Marilena Pipitone, Alessandra Versaci, Nicolò Sella, Marco Lionello, Andy Bertolin

**Affiliations:** 1 Anesthesia and Intensive Care, Vittorio Veneto Hospital, Vittorio Veneto, Italy; 2 Institute of Anesthesiology and Intensive Care, Padua University Hospital, Padova, Italy; 3 Otorhinolaryngology, Vittorio Veneto Hospital, Vittorio Veneto, Italy

**Keywords:** Flow controlled ventilation, FCV, Airway management, Tritube, Laryngeal surgery, Optimized ventilation

## Abstract

**Background:**

Upper airway surgery often poses a challenge to both anesthesiologists and surgeons, as airway access, mechanical ventilation, and surgical difficulties may occur in a tricky combination. To fulfill the need for a tubeless surgery, techniques such as apneic oxygenation or jet ventilation may be used, which carry the risk of several complications. The ultrathin cuffed endotracheal tube Tritube can be used with flow-controlled ventilation (FCV) to provide adequate surgical field and ventilation. To assess the feasibility, safety, and effectiveness of this technique, we describe a series of 21 patients, with various lung conditions, undergoing laryngo-tracheal surgery with FCV delivered via Tritube. Moreover, we perform a narrative systematic review to summarize clinical data on the use of Tritube during upper airway surgery.

**Results:**

All patients were successfully intubated in one attempt with Tritube. The median (interquartile range [IQR]) tidal volume was 6.7 (6.2–7.1) mL/kg of ideal body weight, the median end-expiratory pressure was 5.3 (5.0–6.4) cmH_2_O, and the median peak tracheal pressure was 16 (15–18) cmH_2_O. The median minute volume was 5.3 (5.0–6.4) L/min. Median global alveolar driving pressure was 8 (7–9) cmH_2_O. The median maximum level of end-tidal CO_2_ was 39 (35–41) mmHg. During procedures involving laser, the maximum fraction of inspired oxygen was 0.3, with the median lowest peripheral oxygen saturation of 96% (94–96%). No complications associated with intubation or extubation occurred. In one patient, the ventilator needed to be rebooted for a software issue. In two (10%) patients, Tritube needed to be flushed with saline to remove secretions. In all patients, optimal visualization and accessibility of the surgical site were obtained, according to the surgeon in charge. Thirteen studies (seven case reports, two case series, three prospective observational studies, and one randomized controlled trial) were included in the narrative systematic review and described.

**Conclusions:**

Tritube in combination with FCV provided adequate surgical exposure and ventilation in patients undergoing laryngo-tracheal surgery. While training and experience with this new method is needed, FCV delivered with Tritube may represent an ideal approach that benefits surgeons, anesthesiologists, and patients with difficult airways and compromised lung mechanics.

## Background

The selection of appropriate strategies for managing the airway remains a challenge in patients requiring upper airway surgery. The need to provide adequate oxygenation and carbon dioxide (CO_2_) removal in a secured airway may conflict with the demand for a clean, clear, and spacious surgical field. Moreover, patients undergoing laryngo-tracheal surgery may suffer from increased airway resistance and/or respiratory system elastance, which can require prioritizing adequate ventilation over-optimized surgical conditions.

Several techniques for airway management during laryngo-tracheal surgery, e.g., microlaryngeal tubes (MLTs) and high-frequency jet ventilation (HFJV), have been developed, all providing certain advantages, as well as disadvantages [1, 2].

MLTs are endotracheal tubes with an inner diameter (ID) of 5.0–6.0 mm. Still, in cases of severe tracheal stenosis, the placement of such tubes may be traumatic or even impossible. Furthermore, expiration times might need to be prolonged to avoid overinflation of the lungs, potentially compromising the adequacy of gas exchange, particularly in patients with increased airway resistance and/or respiratory system elastance [3].

HFJV requires the insufflation of oxygen under high pressures through a thin catheter. Exhalation relies on the passive egress of gas and thus demands a patent airway. A “tubeless” HFJV method, i.e., supraglottic superimposed HFJV (SSHFJV), was introduced in the late 90s [4]. Then, surgery is performed through a laryngoscope with integrated jet stream nozzles, enabling ventilation without the need for tracheal intubation. Being supraglottic and tubeless, SSHFJV reduces the risk of barotrauma and fire in case of laser surgery [5, 6]. However, the airway needs to be open to allow a passive egress of gases and to avoid barotrauma. The open airway always carries a risk of aspiration. The jet stream and significant backflow of gases may cause movement of anatomical structures and may generate an aerosol spread [5]. Also, in patients with compromised lung mechanics, SSHFJV may fail because of inadequate ventilation [7].

In situations where the introduction of any tube or catheter would impede the surgical procedure, fully tubeless techniques have been developed. These include the use of HFJV with intermittent phases of apnea, as well as apneic oxygenation with the application of high-flow oxygen [8, 9]. Yet, these methods may result in significant hypercarbia and do not suit those patients with limited apneic window, such as the morbidly obese.

In sum, airway management of upper airway surgical patients with the aforementioned methods may often require the acceptance of suboptimal ventilation and/or compromised surgical conditions.

**Fig. 1:**
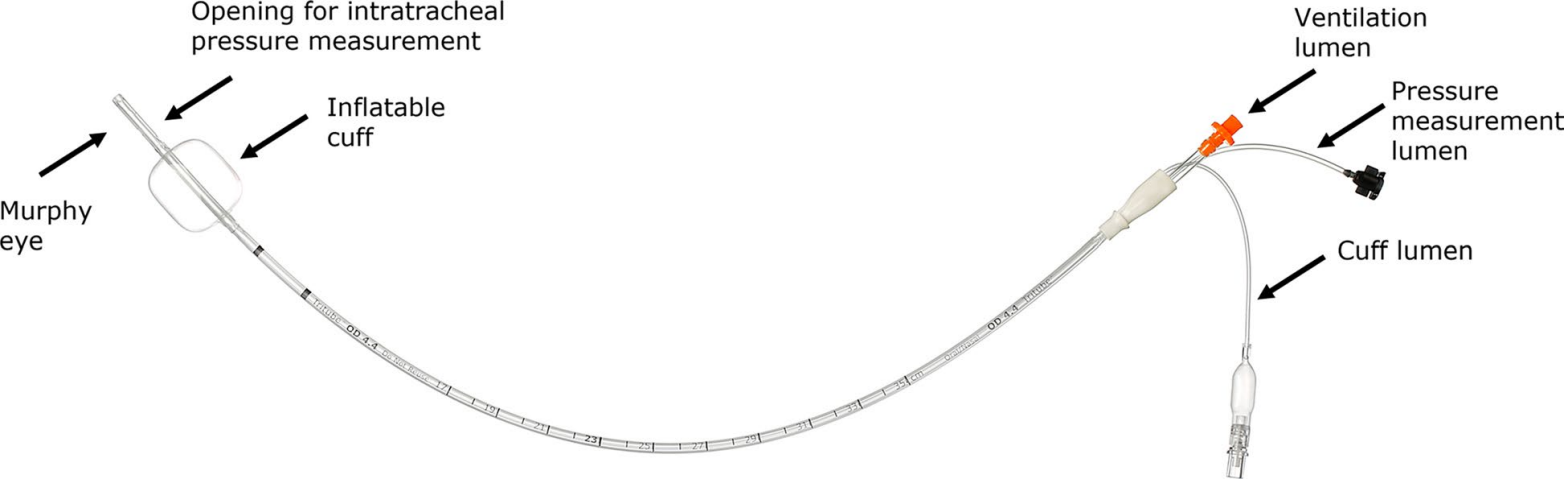
Tritube. Cuffed endotracheal tube with 45-cm length and 4.4-mm outer diameter for adult patient ventilation in combination with FCV. Tritube has three lumens that allow (1) ventilation (2.3-mm inner diameter), (2) inflation and deflation of the cuff, and (3) measurement of intratracheal pressures

**Fig. 2:**
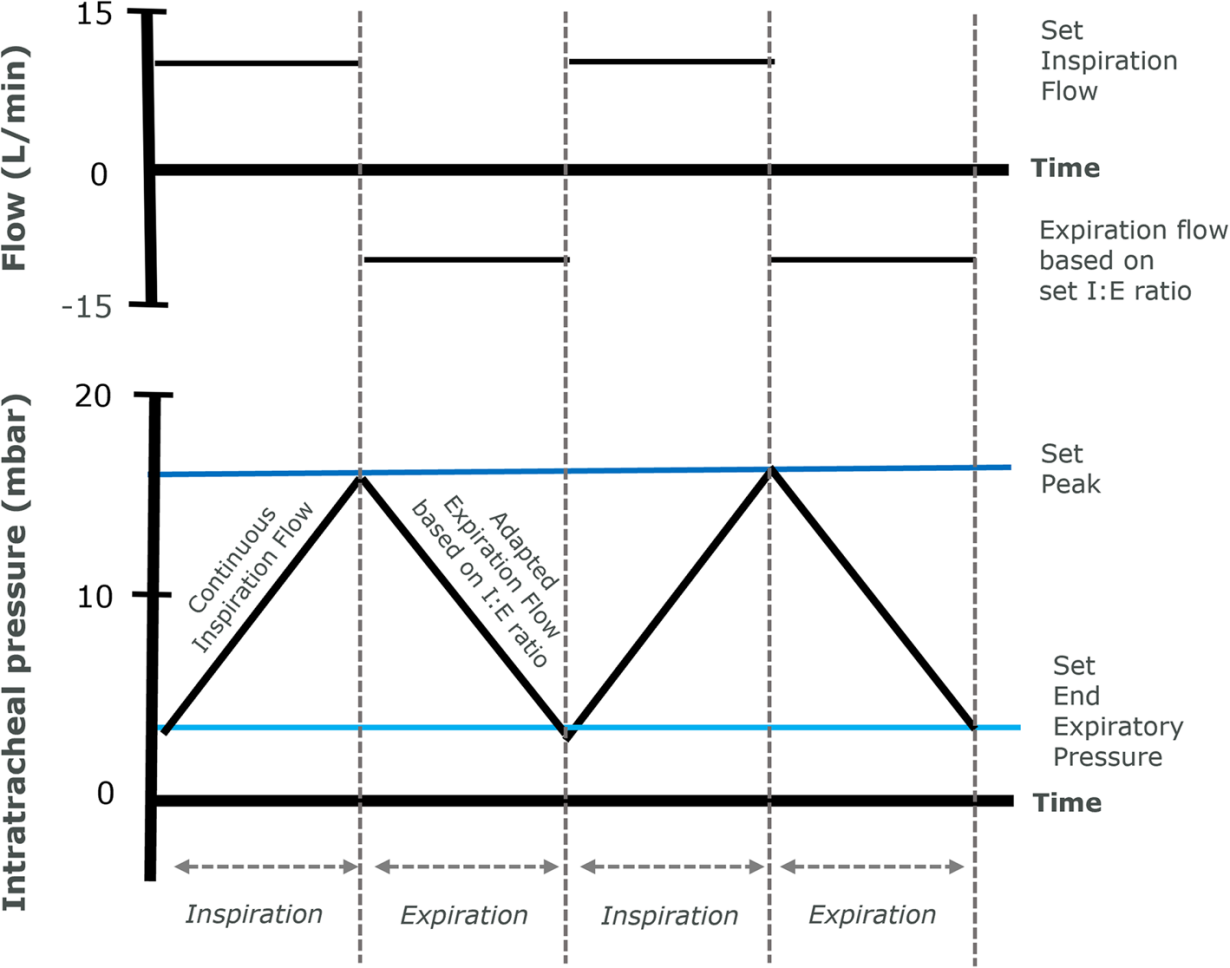
Setting flow-controlled ventilation (FCV). Using FCV requires the setting of four parameters: (1) inspiration flow, (2) I:E ratio, (3) peak pressure, and (4) end-expiratory pressure (EEP). At the set flow rate, gas is insufflated from set EEP until it reaches the set peak pressure. Then, the flow is reversed and gas is sucked out at the rate to reach the set I:E ratio until EEP is reached, aiming for a linear decrease in intratracheal pressure. Then, a next insufflation with the set inspiration flow is started. Applied tidal volume results from the set driving pressure and respiratory system compliance

**Fig. 3:**
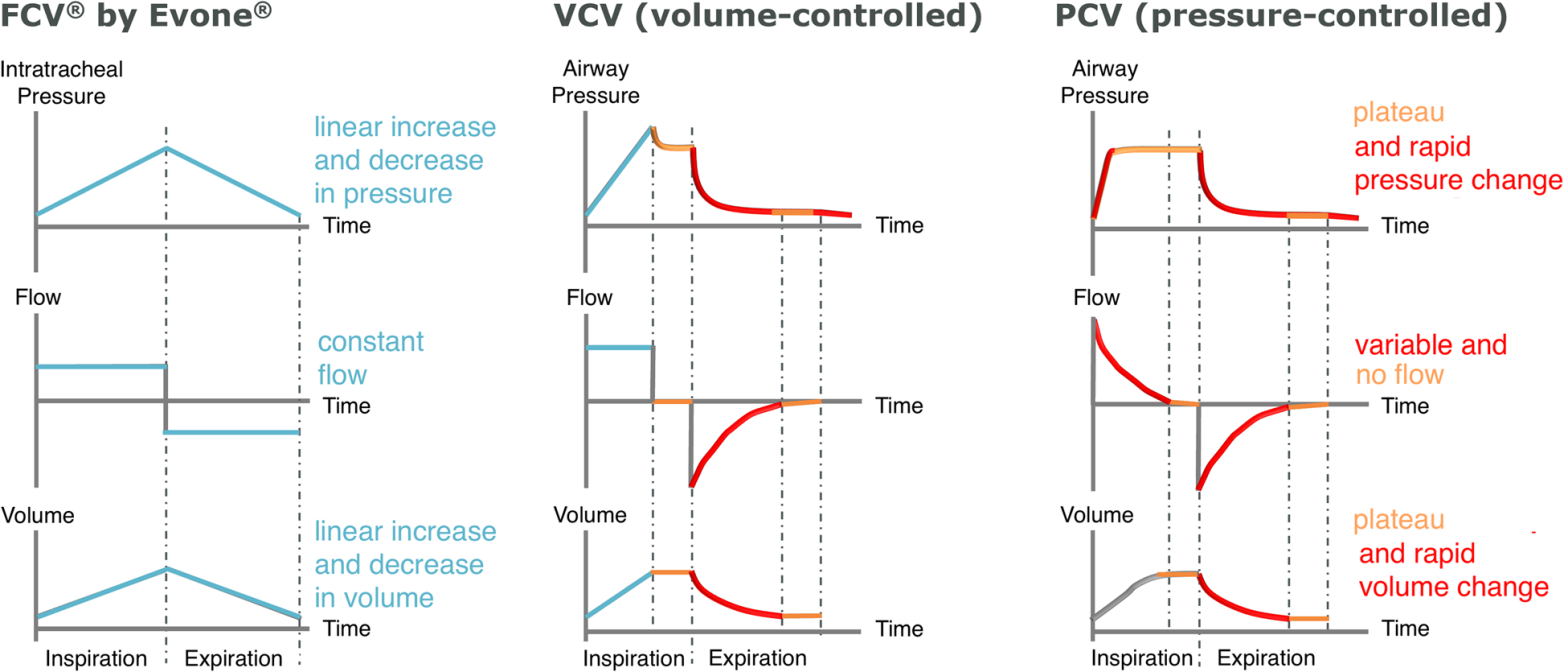
Flow-controlled ventilation (FCV) compared to volume-controlled ventilation (VCV) and pressure-controlled ventilation (PCV). FCV requires a high resistant breathing circuit to prevent passive expiration and to fully control ventilation. FCV is a fully dynamic ventilation providing stable gas flow into and out of the patient lungs, without frequent phases of no-flow. FCV aims for linear increases and decreases in intratracheal pressures, with no sudden pressure drops at the beginning of expiration, and constant flows during inspiration and expiration

The most recent innovation in the field is an ultrathin cuffed endotracheal tube (Tritube®, Ventinova Medical, Eindhoven, the Netherlands), with an ID of 2.3 mm and an outer diameter (OD) of 4.4 mm, that increases surgical exposure, while sealing the airway [10, 11] (Fig. 1). Because of the combination of a cuff sealing the airway and a narrow ID, passive expiration is not possible: the high resistant circuit prevents abrupt and passive deflation of the lungs after inspiration. Therefore, the use of Tritube requires expiration to be actively generated through suctioning. Manual ventilator Ventrain and mechanical ventilator Evone (both Ventinova Medical BV, Eindhoven, the Netherlands) [12–15] provide active expiration by flow-controlled ventilation (FCV). FCV is a ventilatory mode where both inspiratory and expiratory flow rates are maintained constant and low, i.e., < 20 L/min, throughout the respiratory cycle by regulating tracheal pressure, as measured through a dedicated lumen opening at the distal end of the endotracheal tube. Therefore, intratracheal pressure linearly increases and decreases between the chosen end-expiratory pressure (EEP) and peak pressure. During FCV, the inspiratory flow rate, inspiratory to expiratory (I:E) ratio, peak inspiratory pressure, EEP, and FiO_2_ are set by the user, whereas tidal volume and respiratory rate vary depending on ventilator settings and the mechanical properties of the patient’s respiratory system (Fig. 2). Moreover, thanks to accurate mass flow controllers, FCV allows titrating ventilation settings based on measured respiratory system mechanics [16, 17]. The differences between FCV and conventional mechanical modes, i.e., volume-controlled ventilation (VCV) and pressure-controlled ventilation (PCV), regarding the gas flow, tidal volume, and airway pressure waveforms are illustrated in Fig. 3.

We present a retrospective series of 21 patients undergoing laryngo-tracheal surgery with FCV delivered by Tritube. We hypothesize that FCV in combination with Tritube guarantees adequate airway management, surgical exposure, and gas exchange. Also, we perform a narrative systematic review to summarize clinical data on the use of Tritube during upper airway surgery.

## Results

### Patient characteristics

**Fig. 4:**
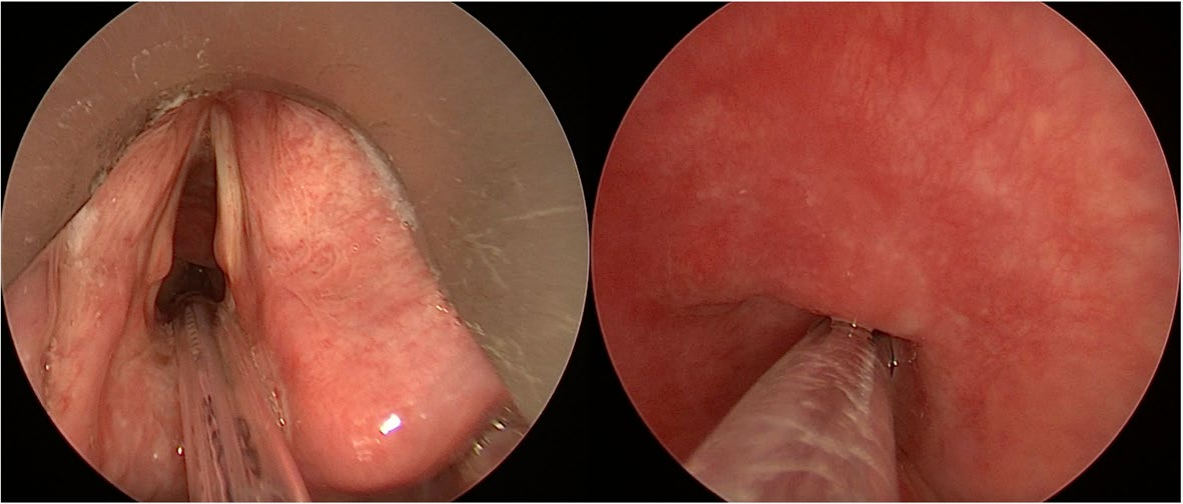
Example of a patient with severe subglottic stenosis intubated with Tritube. Laryngoscopic view above (left) and under (right) the vocal cords

Twenty-one patients with median (interquartile range [IQR]) age of 69 (62–73) years and median American Society of Anesthesiologists (ASA) score of 2 (2–3) are described. Five (24%) patients were obese, four (19%) had severe chronic obstructive pulmonary disease (COPD), and one patient had a history of coronavirus disease (COVID)-19 (Patient #3). All patients underwent laryngo-tracheal surgery with a median duration of 40 (25–50) min and a median ventilation duration of 45 (25–50) min, as indicated in Table 1. Surgical procedures were transoral laser microsurgery (TLM) (*n*=8), cordectomy (*n*=9), microlaryngoscopy for laryngeal bioexeresis (*n*=1), scar toilet after laryngeal TLM (*n*=1), exeresis of glottic synechia (*n*=1), and endoscopic supraglottic laryngectomy (*n*=1). Four (19%) patients had developed severe subglottic/supraglottic stenoses prior to surgery (Fig. 4).

**Table 1:** Patient characteristics and surgical procedures

Patient	Sex (m/f)	Age (years)	BMI (kg/m^**2**^)	PBW (kg)	ASA	Duration of ventilation (min)	Duration of surgery (min)	Surgical procedure
**1**	m	62	24.9	66	2	40	25	Laryngeal bioexeresis microlaryngoscopy
**2**	f	73	26.3	54	2	25	10	Scar toilet after laryngeal TLM
**3**	m	62	25.3	61	3	50	40	TLM for hypoglottic stenosis
**4**	m	64	27	66	2	60	45	Laryngeal biopsy (TLM) for glottic carcinoma
**5**	m	42	27.1	72	2	45	35	Left cordectomy type II
**6**	m	79	30.4	66	2	80	60	Laryngeal biopsy (TLM) for glottic carcinoma
**7**	m	72	31.6	73	2	60	50	Left cordectomy type VI
**8**	f	71	34.5	57	3	45	35	TLM for postoperative neolaryngeal obstruction
**9**	m	62	21.1	70	2	35	25	TLM for suspected laryngeal relapse of carcinoma
**10**	m	57	27.4	70	2	40	25	Left cordectomy type II
**11**	m	69	33.7	69	3	45	35	Exeresis of glottic synechia
**12**	m	78	26.4	68	3	45	40	Endoscopic supraglottic laryngectomy
**13**	m	47	25.6	74	1	15	10	TLM laryngeal granuloma
**14**	f	59	19.4	51	2	35	25	Right cordectomy type II
**15**	f	68	19.7	59	2	40	30	TLM for hypoglottic stenosis
**16**	m	72	19.8	61	3	60	50	TLM for hypoglottic stenosis
**17**	m	69	32.6	70	3	90	80	Right cordectomy type II
**18**	m	75	24.5	70	3	80	65	Right cordectomy type II
**19**	m	73	27.8	73	2	80	60	Right cordectomy type II (revision)
**20**	f	75	19.1	57	2	55	45	Right cordectomy type I
**21**	m	67	27.7	65	2	75	70	Right cordectomy type II
**Median (Q1–Q3)**		**69 (62–73)**	**26 (25–28)**	**66 (61–70)**	**2 (2–3)**	**45 (40–45)**	**40 (25–50)**	

*Abbreviations*: *m* male, *f* female, *BMI* body mass index, *PBW* predicted body weight, *TLM* transoral laser microsurgery, *ASA* American Society of Anesthesiologists score, *Q1* first quartile, *Q3* third quartile

### Airway management

Upon establishment of total intravenous anesthesia (TIVA) (see the “Methods” section), all patients were successfully intubated in one attempt with Tritube by means of videolaryngoscopy. No complications associated with intubation, intraoperative ventilation, or extubation occurred. In two (10%) patients, Tritube needed to be flushed with saline to remove secretions. In all patients, optimal visualization and accessibility of the surgical site were obtained, according to the surgeon in charge. In one case, the adjustments of ventilator settings were suddenly disabled during surgery, despite regularly continuing ventilation, requiring a system reboot. We contacted the manufacturer and the issue was solved in the currently available software.

### Ventilation and gas exchange data

After optimization of FCV ventilation settings (see the “Methods” section), the median tidal volume was 6.7 (6.2–7.1) mL/kg of ideal body weight (IBW) (Table 2), the median EEP was 5 (5–5) cmH_2_O, and median peak tracheal pressure was 16 (15–18) cmH_2_O. The median minute volume was 5.3 (5.0–6.4) L/min. Based on the values of set flow and measured total resistance, global alveolar driving pressure was calculated [18], resulting in a median value of 8 (7–9) cmH_2_O. The median maximum level of end-tidal CO_2_ was 38 (35–41) mmHg. The median lowest peripheral oxygen saturation (SpO_2_) was 96% (94–96%).

**Table 2:** Ventilation data of patients ventilated using Tritube and FCV with individualized settings

Patient	Tidal volume (ml/kg PBW)	Tidal volume (ml)	Respiratory rate (bpm)	Inspiration flow (L/min)	Minute volume (L/min)	Peak pressure (cmH2O)	End expiratory pressure (cmH2O)	Resistance (cmH2O/L/s)	Compliance (mL/cmH2O)	Global alveolar driving pressure (cmH2O)	FiO2	Lower SpO2 (%)	Max etCO2 (mmHg)
**1**	6.2	409	14	12	5.7	12	5	5.1	53	5	0.25	99	35
**2**	5.9	320	16	10	5.1	17	5	12.0	25	8	0.30	94	36
**3**	6.7	410	18	15	7.4	30	1	12.5	13	23	0.30	88	45
**4**	4.1	270	22	12	5.9	18	7	16.2	21	5	0.30	92	43
**5**	6.0	430	14	12	6.0	15	5	12.8	36	5	0.30	96	39
**6**	6.7	440	12	12	5.3	14	5	7.4	46	7	0.30	94	45
**7**	6.7	490	13	12	6.4	15	5	7.6	44	7	0.30	95	45
**8**	6.7	380	16	12	6.1	16	5	8.6	32	8	0.30	96	42
**9**	6.4	447	15	12	6.7	16	5	8.0	37	8	0.25	96	39
**10**	7.5	528	13	13	6.9	15	5	6.5	47	7	0.25	96	41
**11**	6.2	427	17	13	7.3	19	6	10.5	27	8	0.25	93	38
**12**	7.1	480	11	11	5.3	16	5	6.5	41	9	0.25	90	38
**13**	6.2	458	14	12	6.4	13	5	5.9	52	6	0.25	96	38
**14**	7.6	387	9	7	3.5	17	8	10.5	49	7	0.25	96	35
**15**	7.4	410	12	10	4.9	15	5	6.8	39	8	0.30	98	33
**16**	6.4	390	12	9	4.7	18	3	14.7	25	11	0.30	90	36
**17**	6.0	420	12	10	5.0	14	5	8.1	48	7	0.30	96	34
**18**	7.6	531	8	8	3.9	20	5	9.5	37	13	0.30	96	36
**19**	6.2	456	11	10	5.0	18	5	9.6	34	10	0.30	95	34
**20**	9.6	550	7	8	3.5	13	5	7.5	51	6	0.30	98	29
**21**	6.7	452	11	10	5.0	24	9	12.9	29	12	0.30	98	34
**Median (Q1–Q3)**	**6.7 (6.2–7.1)**	**430 (409–458)**	**13 (11–15)**	**12 (10–12)**	**5.3 (5.0–6.4)**	**16 (15–18)**	**5 (5–5)**	**9.6 (7.5–12.4)**	**37 (29–47)**	**8 (7–9)**	**0.30**	**96 (94–96)**	**38 (35–41)**

*Abbreviations*: *PBW* predicted body weight, *SpO2* peripheral oxygen saturation, *etCO2* end-tidal carbon dioxide, *Q1* first quartile, *Q3* third quartile

Twenty (95%) procedures involved laser, requiring lowering the fraction of inspired oxygen (FiO_2_) to 0.3 or lower. In one of these cases, Tritube’s cuff was hit and damaged. Since FCV is most efficient in a sealed airway, this suddenly open airway automatically triggered Evone’s jet ventilation backup mode, which allowed safely completing the case (used settings: respiratory rate 120 bpm, driving pressure 1.5 bar).

### Narrative systematic review

**Fig. 5:**
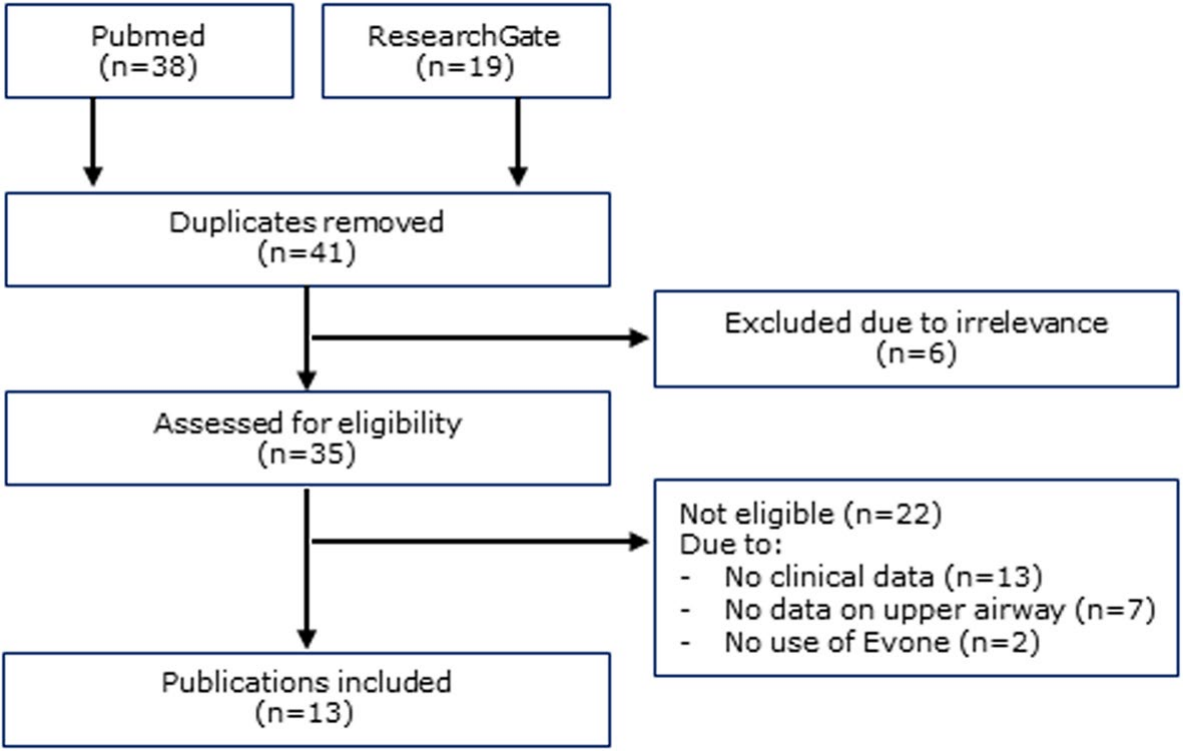
PRISMA flow diagram indicating the citation selection process for the systematic review

After applying the selection criteria (see the “Methods” section), 38 studies were initially identified in PubMed and 19 in ResearchGate. After removing duplicates and applying the exclusion and eligibility criteria, 13 studies were included in the systematic review (Fig. 5): seven case reports, two case series, three prospective observational studies, and one randomized controlled trial (RCT) were selected. Table 3 summarizes these publications.

**Table 3:** Overview and summary of published clinical data using Tritube and FCV in upper airway surgery

Reference	Type	Aim/purpose	Conclusions on Tritube and FCV	Adverse events
Kuut et al (2022) [19]	Prospective observational study (ten patients, data on eight patients reported)	To assess the use of Tritube and FCV in patients with end-to-end anastomosis who had required cross-field intubations in the past.	- Allows intubation in narrowed trachea and adequate ventilation - Avoids the need for cross-field intubation during tracheal anastomosis in most cases - Provides favorable conditions for visual assessment of anastomosis and laryngeal edema following surgery - Primary choice for tracheal surgery	- One cross-field intubation needed due to too weak fixation of Tritube, which was solved after nasal placement of Tritube - One reintubation with another Tritube due to cuff damaged by surgeon. - 14 tube obstructions due to surgical manipulation (six times), secretions (five times), kinking outside of the patient (once), and unknown (twice), which could be solved all times by stopping manipulation or by flushing using saline.
Filauro et al (2022) [20]	Case series (five patients)	Laryngotracheal surgery in five patients, including idiopathic subglottic stenosis (*n*=2) and RRP with subglottic stenosis (*n*=3)	- Can potentially overcome all the drawbacks of HFJV, providing stable and safe ventilation - Increases exposure of the working space for the surgeon - Preserves the airways from bleeding - Protects the surgical team from viral aerosolization during RRP surgery	None
Mallam et al (2022) [21] response by Böttinger et al (2022) [22]	Case report	Management of a near total intrathoracic airway obstruction and its debulking.	- Valuable to manage severe airway obstructions, especially when jet ventilation and ECMO are contra indicated. - Sufficient training is demanded	- Technical errors on Evone (now corrected by software updates) - Ventilatory problems likely related to mispositioning of Tritube - Difficulties in handling Ventrain likely due to the stressful situation
Leow et al. (2022) [23]	Case report	Resection of chondrosarcoma deriving from left arytenoid by means of anterior laryngofissure.	- Allows intubation of the narrowed lumen - Adequate ventilation - Avoids a (temporal) tracheostomy.	None
Bialka et al. (2022) [24]	Case report	Tracheal resection due to severe stenosis after being ventilated due to COVID-19-related ARDS.	- Allows intubation of the narrowed trachea - Provides excellent surgical exposure - Stable and adequate ventilation by FCV - Avoids cross-field intubation	None
Ankay Yilbas et al. (2021) [25]	Case series (three patients)	Three patients undergoing airway surgeries. (1) Emergency debulking surgery and diagnostic biopsy in a patient with a laryngeal mass narrowing the lumen for 80%. (2) Tracheal dilatation in a patient with a history of post-intubation related tracheal resection. (3) Uvulopalatoplasty in an obese patient with severe obstructive sleep apnea.	- Allows intubation in difficult airway - Provides a great surgical exposure - Provides adequate ventilation	Once a short-term obstruction of Tritube with secretions occurred in the third patient, which was resolved after flushing with saline
Bailey et al. (2021) [26]	Case report	Total laryngectomy to resect a stage 4 transglottic squamous cell carcinoma. With a minimum diameter of 2 mm left.	- Permits the surgeons to maintain a closed system during much of the procedure, including during fashioning of the stoma - Avoids need for multiple extubations and periods of apnea - Allows intra-operative assessment of the subglottic tumor - Allows for excellent gas exchange throughout procedure - Avoids awake or emergency tracheostomy	None
Shallik et al. (2021) [27]	Case report	Total thyroidectomy for a malignant, invasive, and highly vascularized thyroid carcinoma that had invaded the surrounding tissues, including the trachea (4-mm-diameter left).	- Allows intubation of a severely narrowed trachea - Provides adequate ventilation using FCV - Avoids the need for ECMO	None
Meulemans et al. (2020) [28]	Prospective observational study (15 patients)	To evaluate feasibility and safety of FCV ventilation using Evone and Tritube in difficult upper airway surgery.	Compared to HFJV, Tritube with FCV: - Allows superior visualization, accessibility and visibility of surgical site - Safe and stable ventilation in all cases - Without the risk of aspiration. - Likely to be time-saving, as it avoids quick desaturation and consecutive surgery pauses.	None
Schmidt et al. (2019) [29]	Randomized controlled trial (2 x 20 patients)	To compare FCV ventilation using Evone and Tritube to VCV using a microlaryngeal tube size 6.0 (MLT-6) in patients without expected difficult airway undergoing elective laryngeal surgery.	- Improves visibility of the surgical site - Improves surgical conditions for users with lower level of expertise - Improves lung aeration and respiratory system compliance	- One Tritube disclocation caused by coughing - One ventilator failure due to software malfunction
Schmidt et al. (2019) [30]	Prospective observational study (15 patients)	To provide assessment of Tritube and FCV in mechanically ventilated lung-healthy patients undergoing ENT surgery	- Allows easy intubation - Contributes to the armamentarium for airway management - Achieves adequate etCO_2_ levels with minute volume and driving pressures in the normal range	- Four tube dislocation (two from coughing, two from external manipulations) - One tube obstruction solved by flushing with saline
Piosik et al. (2018) [31]	Case report	Stepwise synecchia reduction with laser, cold steel instruments and mitomycin C in a patient with severe glottic stenosis upon a history of recurrent laryngeal papillomatosis.	- Facilitates tracheal intubation without compromising the surgical access - Offers prolonged ventilation	None
Jeyarajah and Ahmad (2018) [32]	Case report	Awake intubation to allow panendoscopy in a patient with limited neck extension, a mouth opening of 3 cm, Mallampati score of 3, radiotherapy changes to the neck and COPD.	- Uneventful awake intubation under fiberoptic guidance - Optimal surgical access - Adequate ventilation, with CO_2_ monitoring	None

*Abbreviations*: *FCV* flow-controlled ventilation, *HFJV* high-flow jet ventilation, *RRP* recurrent respiratory papillomatosis, *ECMO* extracorporeal membrane oxygenation, *COVID-19* coronavirus disease-19, *ENT* ear-nose-throat, *etCO2* end-tidal carbon dioxide

The first clinical cases were published in 2018, describing the use of Tritube and Evone in difficult airways and including the use of Tritube for awake intubation [31, 32]. Then, the first prospective observational study reported easy intubation and adequate ventilation in patients undergoing ENT surgery without difficult airway [30]. A direct comparison between Tritube and 6.0-mm-ID MLTs in a randomized controlled trial (RCT), including 40 patients without a difficult airway undergoing elective laryngeal surgery, showed the use of Tritube, as compared to VCV, to result in significantly less concealment of laryngeal structures, improved surgical conditions for less experienced surgeons, better lung aeration, and increased respiratory system compliance [29].

Observational studies including patients with difficult airway showed that Tritube establishes optimal working conditions from laryngeal surgeons’ perspective [28]. It can overcome the drawbacks of jet ventilation, reducing aspiration and contamination risks, while providing adequate ventilation with FCV [20, 28]. Kuut and colleagues showed that, in most cases of tracheal resection, Tritube can avoid the need for cumbersome cross-field intubations [19]. The small OD of Tritube allows stitching the tracheal anastomosis, while keeping the tube in situ and providing continuous ventilation in a sealed airway. Further, Tritube provided good conditions for the visual assessment of the anastomosis and laryngeal edema after surgery.

The value of the combination of Tritube with FCV has been confirmed in several case reports, including the treatment of severe tracheal stenoses and laryngectomies, where Tritube allowed (awake) intubation of narrow airways, therewith avoiding periods of apnea, cross-field intubation, provisional tracheostomy or extracorporeal membrane oxygenation (ECMO) [21, 23–27, 32]. Also, considering the reduced aerosol spreading, laryngeal papillomatosis may be another indication for preferring Tritube over HFJV [20, 31].

In total, data on 75 patients ventilated through Tritube were reported in 13 publications. In five publications, adverse events occurred. Tube obstruction was the most frequently reported event, which happened 16 times in eight patients (of which six undergoing tracheal resection). Obstruction was caused by surgical manipulation (six times), secretions (seven times), kinking outside of the patient (once), or unknown reasons (twice). In all cases, secretions could be removed by flushing the lumens with saline. Tube dislocation was reported six times and was due to surgical manipulation (three times) or coughing (three times). Dislocation by coughing was only reported in the early studies. Tritube’s relatively big cuff, due to its small lumen, and the high resistant breathing circuit generate the risk for dislodgement upon coughing. These publications likely made users more aware of the importance of optimizing the depth of anesthesia in order to avoid coughing and spontaneous breathing efforts. Cuff damage was reported only once, upon surgical manipulations. Tritube had to be replaced, which went uneventful. In two cases, the Evone ventilator caused ventilation difficulties. In both cases, a software update solved the issue [21, 29].

No direct clinical comparisons between SSHFJV and FCV were published.

## Discussion

In this series of 21 patients with various lung comorbidities undergoing laryngo-tracheal surgery for different indications, we show that Tritube in combination with FCV provides good surgical conditions and adequate gas exchange at relatively low minute volumes and global alveolar driving pressures. Our results are in line with earlier publications and suggest this technique to be feasible even in patients with compromised lung mechanics.

Current strategies for airway management during upper airway surgery often require the acceptance of risks related to anesthetic and/or surgical considerations. In contrast, the use of FCV delivered by Tritube may preserve surgical view, while allowing airway protection and adequate gas exchange. First, in our patients, the small OD of Tritube (4.4 mm) allowed easy intubation, even in case of tracheal stenosis, and a spacious working field for the surgeon. Second, no aspiration event occurred, thus minimizing the risk of aspiration pneumonia and atelectasis and aiding in achieving a clean surgical environment. Third, no passive backflow of gases occurred, hence reducing aerosol spread and improved surgical view. This last advantage was recognized by experts in the field of airway management, who recently published recommendations aiming at minimizing the risk of aerosol spread in the setting of upper airway surgery during the COVID-19 pandemic [2]. Furthermore, according to the recent description of surgical tracheotomy using Tritube and FCV, these techniques could increase the safety of patients and medical personnel by avoiding the need for cumbersome cross-field intubations and reducing the aerosol generation, respectively [33]. Fourth, Tritube may be less rigid and traumatic than laser-resistant tubes. While laser resistance is not claimed for Tritube by the manufacturer, we did use it during laser surgery by protecting the cuff with saline-soaked gauze as described earlier [20, 28], which, being located distally to the lesion, did not compromise the surgical exposure. The FiO_2_ could be easily reduced to 0.3 or lower in our patients.

The practical advantages of Tritube with respect to airway management and surgical conditions are combined with several ventilation benefits.

First, when compared to VCV or PCV, the relatively low and constant inspiratory and expiratory flow during FCV may result in more homogenous lung aeration and recruitment, better gas exchange, and higher ventilation efficiency [15, 29, 34–38]. Moreover, as compared with HFJV, FCV reduces the risk of air-trapping, hyperinflation, and associated barotrauma [28, 39, 40].

Second, since Tritube enables intratracheal pressure measurements and Evone utilizes mass flow controllers, respiratory system mechanics can be precisely determined [41, 42]. This offers the possibility to individually titrate applied flow and pressure based on the dynamic compliance of the patient’s respiratory system [16, 43]. This approach might reduce driving pressure, i.e., the difference between plateau pressure and positive end-expiratory pressure and mechanical power, i.e., the total energy transferred from the mechanical ventilator to the lungs during inflation [17, 42]. In our study, we observed adequate gas exchange and acceptable driving pressures in all patients, except for a post-COVID-19 patient (patient #3) with severely impaired respiratory system mechanics. In this patient, a very high global alveolar driving pressure (23 cmH_2_O) was required to achieve acceptable oxygenation and normocapnia, while using a minute volume of only 7.4 L/min. In a patient with COVID-19 acute respiratory distress syndrome (ARDS) in the intensive care unit (ICU), Spraider et al. recently found FCV to improve oxygenation, as compared to PCV, while reducing the applied energy of ventilation [43]. Recently, in a small crossover study in 10 patients with COVID-19 ARDS, our group found lower mechanical power and higher ventilation efficiency during FCV compared to VCV [44]. Another recent crossover study comparing FCV with VCV also observed improved ventilation efficiency by FCV [45].

Our study has some limitations. First, the small sample size of our retrospective single-center case series makes our findings exploratory and hypothesis-generating. Importantly, we did not compare FCV delivered by Tritube with other airway management strategies in our patients. Further prospective comparative studies with larger sample sizes are needed to confirm our findings. Second, the achievement of adequate gas exchange could not be confirmed by arterial blood gas analyses, as invasive arterial pressure was not part of our routine intraoperative monitoring. Third, no quantitative assessment of surgical exposure was reported nor imaging aiming at evaluating lung aeration was performed. Fourth, this series of cases was performed by one anesthesiologist (AG), who has 15 years of experience, of which many in airway surgery procedures. The determination of a learning curve was out of scope of this study. Though, in order to gain adequate confidence with the technique, we suggest a minimum of 10 supervised operations.

Some limitations pertaining to FCV and Tritube should also be addressed.

First, FCV represents a novel ventilator mode, requiring specific training.

Second, training is also recommended for the management of Tritube. The small ID of Tritube makes it more susceptible to obstruction by secretions, which may affect or even interrupt ventilation. Therefore, a “plan B” (e.g., hand-held ventilator Ventrain) is always needed, but proper preparation on the forehand can significantly reduce the risk on obstructions. First, it is important to judge on the probability of secretions (e.g., COPD, secretion retention due to obstruction) and to remove these where possible by asking the patient to cough before anesthetizing and to suck away secretions before intubation. Others described to administer glycopyrronium to reduce mucosal secretions [28]. During intubation, Tritube is advanced from its stylet further down the trachea while turning. When the tube is at the right position, it should be slightly pulled back before inflating the cuff to free Tritube’s tip from the tracheal wall. Then, one can flush both the ventilation lumen and pressure lumen with air to ensure the absence of any obstructions. Upon starting FCV ventilation, the machine will also perform a purge with air automatically. During ventilation, irregular pressure curves may indicate the presence of secretions (but may also result from light anesthesia or mispositioning of Tritube). An altered shape of the pressure curve without any other explanation may be solved by flushing the pressure and ventilation lumens using 2–5 cc of saline followed by 15 cc of air. Should the issue persist, one may consider to use the suction catheter to remove secretions from the ventilation lumen after deflating the cuff should be deflated. As mentioned, a mispositioning of Tritube may also affect the shape of the pressure curve. The tube is 45 cm long and may touch the carina or migrate into a bronchus because of surgical manipulations. Furthermore, the higher flexibility of Tritube, when compared to reinforced laser-resistant tubes, makes it more prone to compression during suspension laryngoscopy. Complete obstruction of one of the lumens will be detected by the ventilator, that responds by alarming while purging the lumens. Also, one may use the Jet ventilation mode to purge away an obstruction with a deflated cuff.

Third, as mentioned earlier, Tritube is not laser-safe and its use during laser surgery is not intended by the manufacturer. Indeed, we report the Tritube’s cuff damage during one case. However, given its small OD and the resulting unimpeded surgical view, no complications during laser surgery have been reported by other authors, provided that FiO_2_ is lowered and the tube and/or its cuff are covered with a wet gauze [20, 28].

Fourth, patients ventilated with FCV should be kept under TIVA with muscle relaxation throughout the procedure to avoid spontaneous breathing efforts and coughing, which may lead to Tritube dislocation and interruption of ventilation [30].

## Conclusion

In 21 patients undergoing laryngo-tracheal surgery, we observed that the utilization of the ultrathin and cuffed Tritube in combination with FCV allowed easy intubation, good surgical exposure, low chance of aerosol spread, and adequate gas exchange. Like any other new technique, this method requires device-specific training to get adequate confidence. Our observations add to the growing body of evidence that Tritube and FCV may represent valuable strategies for airway management in laryngo-tracheal surgery, even in patients with lung comorbidities (e.g., post-COVID-19, COPD) and severe tracheal stenosis.

## Methods

### Aim, design, and setting

This study is a single-center retrospective observational study. Twenty-one consecutive patients scheduled for laryngo-tracheal surgery were included between November 2020 and June 2021.

The decision to use Evone and Tritube (both Ventinova Medical BV, Eindhoven, the Netherlands) was made by the anesthesiologist and surgeon in charge based on the indication for treatment. The study was approved by the Local Ethical Committee (Comitato Etico di Sperimentazione Clinica ULSS 2 Marca Trevigiana, Prot. 96489, 19/05/2022) and was conducted in accordance with the principles of the Helsinki Declaration.

### Anesthesia

Following adequate pre-oxygenation, TIVA was induced with intravenous propofol and remifentanil, targeting a bispectral index of 40–60 during surgery. Upon induction, patients received fentanyl 2 μg/kg and rocuronium 0.6 mg/kg for neuromuscular blockade with target train-of-four (TOF) of 0 to 1. For decurarization, sugammadex 2 mg/kg was administered based on TOF to minimize the time frame between the initial triggering of the patient and spontaneous breathing allowing extubation.

### Intubation and extubation

Tritube is a cuffed endotracheal tube made of polyurethane (length 45 cm, OD 4.4 mm) and has three lumens: a ventilation lumen conducting inspiratory and expiratory gas flow, a pressure measurement lumen allowing measurements of intratracheal pressures, and a cuff lumen to inflate and deflate the low pressure/high volume cuff (Fig. 1).

Tritube was positioned using videolaryngoscopy (C-MAC, Karl Storz Endoskope®, Tuttlingen, Germany) and gently rotated while directed through the vocal cords. We believe that the rotation of the tip of the tube is particularly important in the case of subglottic stenosis. Since the cuff of the tube is relatively large as compared to its OD, it may compromise the view during intubation. Therefore, the cuff was wrapped around the tube before intubation, which was done by simultaneously rotating the tube covered with a gauze while deflating the cuff.

Upon any occurring obstruction of Tritube, e.g., by secretions, the ventilation and pressure lumens were flushed with saline and/or air.

In patients scheduled for laser-assisted treatment, adequate precautions were taken to avoid damage of Tritube and/or its cuff and to reduce the risk of airway fires. The applied FiO_2_ was reduced to 0.3, while the cuff of Tritube was covered with wet gauze (cotonoid strip).

As soon as the patient showed the first signs of triggering, the cuff of Tritube was deflated immediately and oxygen insufflation through Tritube was maintained until the patient was ready for extubation.

### Ventilation

Upon establishment of TIVA and intubation, Tritube was connected to mechanical ventilator Evone, and FCV was initiated at default settings. Then EEP and peak pressure were optimized based on measured lung mechanics as described below, while keeping the tidal volume around 6 mL/kg of IBW. The inspiratory flow was adjusted to achieve a minute ventilation allowing normal etCO_2_.

### FCV setting optimization

Individualization of FCV was based on a previously described method [16]. EEP was stepwise increased or decreased while keeping the driving pressure constant until the highest tidal volume/ compliance was reached. Then, peak pressure was stepwise increased or decreased until the highest compliance was reached, while keeping the tidal volume around 6 mL/kg of IBW.

Finally, the inspiratory flow was adjusted to achieve normocapnia at an I:E ratio of 1:1, which is considered the best for minimizing dissipated energy [17].

### Dynamic FCV and global alveolar driving pressure

During FCV, because of the patient’s airway resistance, tracheal pressures measured and displayed by the ventilator deviate to a certain extent from the actual alveolar pressures [41]. As a result, the global alveolar driving pressure is typically lower than the tracheal driving pressure calculated from measured intratracheal EEP and peak values, while the calculated dynamic compliance is underestimated [18]. The extent of these deviations is both flow- and resistance-dependent: the higher the set flow and the airway resistance during FCV, the greater the difference between tracheal and global alveolar driving pressures. As the difference in pressures is stable during the entire ventilation cycle, accurate calculations of alveolar driving pressure can be easily performed based on given flow and measured airway resistance [18]. First, the pressure needed to overcome the total resistance (mbar) is calculated by dividing the measured total resistance (mbar/L/s) by the set inspiratory low (L/s). Then, based on the measured dynamic intratracheal peak pressure (mbar) and EEP (mbar), the mean global alveolar pressures can be calculated, as follows: mean global alveolar peak pressure (mbar) = dynamic intratracheal peak pressure (mbar) − pressure needed to overcome resistance (mbar); mean global alveolar EEP (mbar) = dynamic intratracheal EEP (mbar) + pressure needed to overcome resistance (mbar). These calculations assume an I:E ratio of 1.0:1.0, leading to similar flows during inspiration and expiration, and that the resistance measured at peak pressure is similar to that measured at EEP. For convenience, the manufacturer provided a tool to easily perform these calculations and provided a table with pre-calculated values for a quick estimation (Table 4)

**Table 4:** Pre-calculated pressure differences between dynamic tracheal and dynamic global alveolar pressures for various set flows and measured resistances, as provided by the manufacturer

Measured total resistance (mbar/L/s)			Difference (mbar) P_**trachea**_ vs P_**alveolar**_ (Peak_**trach**_ > Peak_**alv**_ and PEEP_**trach**_ < PEEP_**alv**_)								
			5	10	15	20	25	30	35	40	50
Flow (L/s)		**Flow (L/min)**									
0.133	=	**8**	1	1	2	3	3	4	5	5	7
0.167	=	**10**	1	2	3	3	4	5	6	7	8
0.200	=	**12**	1	2	3	4	5	6	7	8	10
0.233	=	**14**	1	2	4	5	6	7	8	9	12
0.267	=	**16**	1	3	4	5	7	8	9	11	13
0.300	=	**18**	2	3	5	6	8	9	11	12	15
0.333	=	**20**	2	3	5	7	8	10	12	13	17

### Surgery

Various surgical upper airway procedures were performed, using a rigid laryngoscope in all cases. Most of the procedures (20/21) used the operating microscope Leica F40 (Leica Microsystems Srl, Milan, Italy) and the CO_2_ laser Lumenis Ultra PulseDuo (Boston Scientific, Marlborough, MA, USA).

### Clinical data collection

Data were retrospectively retrieved from the electronic medical files. For all patients, a review of relevant medical history and anesthesia and surgical reports was performed. Twenty parameters (age, gender, body mass index, ASA Physical Status Classification system, duration of ventilation, duration of surgery, surgical procedure, tidal volume, respiratory rate, peak pressure, EEP, inspiratory flow, minute volume, airway resistance, dynamic respiratory system compliance, global alveolar driving pressure [18], FiO_2_, highest etCO_2_, lowest SpO_2_, and complications related to the use of Tritube and Evone) were collected.

### Systematic narrative review

We searched PubMed since inception until July 29, 2022, using the following text words: “tritube” OR “flow controlled ventilation” OR (“narrow bore lumen” AND “mechanical ventilation”). Then, we searched ResearchGate using the text word “tritube”. All duplicates and irrelevant publications were removed. The following eligibility criteria were applied: (1) studies containing clinical data, (2) studies on upper airway surgery, and (3) studies on the use of FCV by mechanical ventilator Evone.

## Data Availability

The datasets used and/or analyzed during the current study are available from the corresponding author on reasonable request.

## Abbreviations

ARDS: Acute respiratory distress syndrome
ASA: American Society of Anesthesiologists
BMI: Body mass index
COVID-19: Coronavirus disease-19
EEP: End-expiratory pressure
ENT surgery: Ear, nose, and throat surgery
etCO_2_: End-tidal carbon dioxide
FCV: Flow-controlled ventilation
FiO_2_: Fraction of inspired oxygen
HFJV: High-frequency jet ventilation
ID: Inner diameter
MLT: Microlaryngeal tube
OD: Outer diameter
PCV: Pressure-controlled ventilation
SSHFJV: Supraglottic superimposed high-frequency jet ventilation
SpO_2_: Peripheral oxygen saturation
TIVA: Total intravenous anesthesia
TLM: Transoral laser microsurgery
VCV: Volume-controlled ventilation

## References

[1] Edelman DA, Perkins EJ, Brewster DJ (2019). Difficult airway management algorithms: a directed review. Anaesthesia.

[2] Nouraei SAR, Girgis M, Shorthouse J, El-Boghdadly K, Ahmad I (2020). A multidisciplinary approach for managing the infraglottic difficult airway in the setting of the coronavirus pandemic. Oper Tech Otolaryngol Head Neck Surg.

[3] Gemma M (2016). Intrinsic positive end-expiratory pressure during ventilation through small endotracheal tubes during general anesthesia: incidence, mechanism, and predictive factors. J Clin Anesth.

[4] Ihra G (1999). Supralaryngeal tubeless combined high-frequency jet ventilation for laser surgery of the larynx and trachea. Br J Anaesth.

[5] Lanzenberger-Schragl E, Donner A, Grasl MC, Zimpfer M, Aloy A (2000). Superimposed high-frequency jet ventilation for laryngeal and tracheal surgery. Arch Otolaryngol Head Neck Surg.

[6] Rezaie-Majd A (2006). Superimposed high-frequency jet ventilation (SHFJV) for endoscopic laryngotracheal surgery in more than 1500 patients. Br J Anaesth.

[7] Halmos GB (2020). Predictors for failure of supraglottic superimposed high-frequency jet ventilation during upper airway surgery in adult patients; a retrospective cohort study of 224 cases. Clin Otolaryngol.

[8] Youssef DL, Paddle P (2021). Tubeless anesthesia in subglottic stenosis: comparative review of apneic low-flow oxygenation with THRIVE. Laryngoscope.

[9] Ly NM, Van Dinh N, Trang DTT, Hai NV, Hung TX (2022). Apnoeic oxygenation with high-flow oxygen for tracheal resection and reconstruction surgery. BMC Anesthesiol.

[10] Enk D (2018). Jet-Ventilationskatheter, insbesondere zur Beatmung eines Patienten (jet ventilation catheter, in particular for ventilating a patient). United States Patent n US.

[11] Kristensen MS, de Wolf MWP, Rasmussen LS (2017). Ventilation via the 2.4 mm internal diameter Tritube® with cuff - new possibilities in airway management. Acta Anaesthesiol Scand.

[12] Enk, D. Verfahren und Vorrichtung zur Beatmung eines Patienten (method and device for ventilating a patient), Canadian Patent Application CA3016247A1 (2017) https://patents.google.com/patent/CA3016247A1.

[13] Enk D (2020). Gasstromumkehrelement (gas flow reversing element). United States Patent n US.

[14] Hamaekers AEW, Borg PAJ, Enk D (2012). Ventrain: an ejector ventilator for emergency use. Br J Anaesth.

[15] Schmidt J (2018). Improved lung recruitment and oxygenation during mandatory ventilation with a new expiratory ventilation assistance device: a controlled interventional trial in healthy pigs. Eur J Anaesthesiol.

[16] Spraider P (2020). Individualized flow-controlled ventilation compared to best clinical practice pressure-controlled ventilation: a prospective randomized porcine study. Crit Care.

[17] Barnes T, van Asseldonk D, Enk D (2018). Minimisation of dissipated energy in the airways during mechanical ventilation by using constant inspiratory and expiratory flows - flow-controlled ventilation (FCV). Med Hypotheses.

[18] Enk D, Abram J, Spraider P, Barnes T (2021). Dynamic compliance in flow-controlled ventilation. Intensive Care Med Exp.

[19] Kuut MH, Honings J, Marres HAM, Mourisse JMJ, Verhagen AFTM (2022) Controlled mechanical ventilation through a narrow bore lumen during tracheal surgery: a prospective observational study. Eur J Anaesthesiol. 10.1097/EJA.0000000000001717 10.1097/EJA.0000000000001717 35875915

[20] Filauro M (2022). Evone® flow controlled ventilation: a new device for laryngotracheal surgery. Acta Otorhinolaryngol Ital.

[21] Mallam L, Massingberd-Mundy D, Girgis M, De Zoysa N (2022) Near total intrathoracic airway obstruction managed with a Tritube® and flow-controlled ventilation. Anaesth Rep 10. 10.1002/anr3.12156 10.1002/anr3.12156 PMC8885748 35252872

[22] Böttinger L, Uriarte J, van der Hoorn JWA (2022). Near total intrathoracic airway obstruction managed with a Tritube (R) and flow-controlled ventilation: a reply. Anaesth Rep.

[23] Leow TYS, Van der Wal RAB, Marres HAM, Honings J (2022). Intubation with a TriTube to avoid peri-operative tracheostomy in open airway surgery. J Laryngol Otol.

[24] Bialka S (2022). Flow-controlled ventilation - a new and promising method of ventilation presented with a review of the literature. Anaesthesiol Intensive Ther.

[25] Yilbas AA (2021). Experience with Tritube and flow-controlled ventilation during airway surgery. Turk J Anaesthesiol Reanim.

[26] Bailey JR (2021). Laryngectomy with a Tritube® and flow-controlled ventilation. Anaesth Rep.

[27] Shallik N et al (2021) Management of critical tracheal stenosis with a straw sized tube (Tritube): case report. Qatar Med J 2020(3):48. 10.5339/qmj.2020.48 10.5339/qmj.2020.48 PMC7842837 33598418

[28] Meulemans J (2020). Evone® flow-controlled ventilation during upper airway surgery: a clinical feasibility study and safety assessment. Front Surg.

[29] Schmidt J (2019). Glottic visibility for laryngeal surgery: Tritube vs. microlaryngeal tube: a randomised controlled trial. Eur J Anaesthesiol.

[30] Schmidt J (2019). Flow-controlled ventilation during ear, nose and throat surgery: a prospective observational study. Eur J Anaesthesiol.

[31] Piosik ZM, Todsen T, Balle JS, Abildstrøm H, Kristensen MS (2018). Ultra-narrow 2.4 mm id Tritube® together with Evone® ventilation allows surgical access and controlled ventilation even in case of severe stenosis. Trends Anaesth Crit Care.

[32] Jeyarajah K, Ahmad I (2018). Awake tracheal placement of the Tritube® under flexible bronchoscopic guidance. Anaesth Rep.

[33] Magasich-Airola NP, Rosal Martins M, Desuter GR, Van Boven MJ (2021). Novel technique for safe tracheostomy during COVID-19 pandemic using Evone® flow-controlled ventilation system. Int J Clin Pract.

[34] Weber J (2020). Flow-controlled ventilation (FCV) improves regional ventilation in obese patients – a randomized controlled crossover trial. BMC Anesthesiol.

[35] Schmidt J (2020). Flow-controlled ventilation attenuates lung injury in a porcine model of acute respiratory distress syndrome: a preclinical randomized controlled study. Crit Care Med.

[36] Wenzel C (2020). A linearized expiration flow homogenizes the compartmental pressure distribution in a physical model of the inhomogeneous respiratory system. Physiol Meas.

[37] Sebrechts T, Morrison SG, Schepens T, Saldien V (2021). Flow-controlled ventilation with the Evone ventilator and Tritube versus volume-controlled ventilation: a clinical cross-over pilot study describing oxygenation, ventilation and haemodynamic variables. Eur J Anaesthesiol.

[38] Weber J, Schmidt J, Straka L, Wirth S, Schumann S (2020). Flow-controlled ventilation improves gas exchange in lung-healthy patients— a randomized interventional cross-over study. Acta Anaesthesiol Scand.

[39] Wirth S, Seywert L, Spaeth J, Schumann S (2016). Compensating artificial airway resistance via active expiration assistance. Respir Care.

[40] Paxian M, Preussler NP, Reinz T, Schlueter A, Gottschall R (2015). Transtracheal ventilation with a novel ejector-based device (Ventrain) in open, partly obstructed, or totally closed upper airways in pigs. Br J Anaesth.

[41] Enk D, Spraider P, Abram J, Barnes T (2020). Pressure measurements in flow-controlled ventilation. Crit Care Med.

[42] Barnes T, Enk D (2019). Ventilation for low dissipated energy achieved using flow control during both inspiration and expiration. Trends Anaesth Crit Care.

[43] Spraider P (2021). A case report of individualized ventilation in a COVID-19 patient – new possibilities and caveats to consider with flow-controlled ventilation. BMC Anesthesiol.

[44] Grassetto, A. et al. Flow-controlled ventilation may reduce mechanical power and increase ventilatory efficiency in severe coronavirus disease-19 acute respiratory distress syndrome. at 10.21203/rs.3.rs-1438128/v1 (2022). 10.1016/j.pulmoe.2022.05.006 PMC9186429 35864057

[45] Van Dessel ED, De Meyer GR, Morrison SG, Jorens PG, Schepens T (2022). Flow-controlled ventilation in moderate acute respiratory distress syndrome due to COVID-19: an open-label repeated-measures controlled trial. Intensive Care Med Exp.

